# Transcriptomics and metabolomics changes triggered by exogenous 6-benzylaminopurine in relieving epicotyl dormancy of *Polygonatum cyrtonema* Hua seeds

**DOI:** 10.3389/fpls.2022.961899

**Published:** 2022-07-25

**Authors:** Wenwu Zhang, Long Xia, Fulei Peng, Chenyu Song, Muhammad Aamir Manzoor, Yongping Cai, Qing Jin

**Affiliations:** School of Life Sciences, Anhui Agricultural University, Hefei, China

**Keywords:** *Polygonatum cyrtonema* Hua seeds, epicotyl dormancy, transcriptomic, metabolomic, co-expression analysis

## Abstract

*Polygonatum cyrtonema* Hua is one of the most useful herbs in traditional Chinese medicine and widely used in medicinal and edible perennial plant. However, the seeds have the characteristics of epicotyl dormancy. In this study, the molecular basis for relieving epicotyl dormancy of *P. cyrtonema* seeds under exogenous 6-benzylaminopurine (6-BA) treatment was revealed for the first time through transcriptome and metabolomics analysis. We determined the elongation of epicotyl buds as a critical period for dormancy release and found that the content of trans-zeatin, proline, auxin and gibberellin was higher, while flavonoids and arginine were lower in the treatment group. Transcriptome analysis showed that there were significant differences in gene expression in related pathways, and the expression patterns were highly consistent with the change of metabolites in corresponding pathways. Co-expression analysis showed that cytokinin dehydrogenase of *P. cyrtonema* (*PcCKXs*) and pelargonidin in flavonoid biosynthesis, as well as L-proline, L-ornithine, and L-citrulline in arginine and proline metabolism form network modules, indicating that they have related regulatory roles. Above all, our findings provide new insight into the exogenous 6-BA relieving epicotyl dormancy of *P. cyrtonema* seeds.

## Introduction

*Polygonatum cyrtonema* Hua (*P. cyrtonema*) is a medicinal food homology perennial plant (Huang et al., 2018; Wang et al., 2019), which is distributed in the northern hemisphere temperate regions, such as China, Russia, Europe, and North America (Li et al., 2015; Liu et al., 2017; Zhao et al., 2018). It is an important traditional Chinese herb for treating diabetes and asthma (Ma et al., 2020) and contains flavonoids and polysaccharides, which have the effects of anti-fatigue and treatment of diabetes (Wdsa et al., 2021).

*Polygonatum macranthum* seeds have the characteristics of “double dormancy,” that is, radicle dormancy and epicotyl dormancy (Lekamge et al., 2020). The epicotyl dormancy is that when the seed dormancy is released, the radicle breaks through the seed to germinate, and then continues to grow and gradually develop into a corm. After that, *P. cyrtonema* seeds enter dormancy again. Therefore, under natural conditions, *P. cyrtonema* seeds need to undergo two winters to emerge smoothly, and the breeding cycle is very long, which greatly limits the large-scale planting. Plant hormones play an important role in plant dormancy release. And cytokinin can promote cell division, bud differentiation and break dormancy (Kieber and Schaller, 2014). 6-Benzylaminopurine (6-BA), the first synthetic cytokinin, is commonly used to relieve dormancy in plants. Adding exogenous 6-BA could relieve the dormancy of Danfeng seeds breaking the epicotyl (Ren et al., 2019) and promotes the growth of apple axillary buds (Tan et al., 2019). Meanwhile, *CKX*, a cytokinin oxidase gene, regulates plant cytokinin homeostasis by degrading cytokinin (Liu L. M. et al., 2021). Abscisic acid (ABA) promotes the induction and maintenance of dormancy and inhibits seed germination (Sano and Marion-Poll, 2021), while cytokinin and ABA show antagonistic effects (Huang et al., 2018).

In addition, other metabolites also play an important role in dormancy release. Research shows that SlAN11 regulates flavonoid biosynthesis and seed dormancy by interacting with bHLH protein in tomato (Gao et al., 2018). Arginine content has a significant effect on dormancy release. Previous studies have reported that arginine regulates seed dormancy and its dephosphorylation promotes seed germination (Zhou et al., 2019). Proline contributes to osmotic balance between cytoplasm and vacuole, and the relative level of free proline in apple bud is higher. Proline contributes to the osmotic balance between cytoplasm and vacuole. The relative level of free proline in grape buds is higher (Mujahid et al., 2020), which indicates that proline promotes dormancy release.

Seeds of *Polygonatum cyrtonema* have characteristics of epicotyl dormancy (Feilong et al., 2017). This has seriously affected the cultivation of *Polygonatum cyrtonema*. Epicotyl dormancy is a type of dormancy that the radicle develops into roots and the hypocotyls do not elongate, and shoot emergence always delay after radicle protrusion (Yang et al., 2017). Seed dormancy can be relieved by low temperature treatment (Zhang K. et al., 2019), and 6-BA can also make *P. cyrtonema* emergence but 0–500 mg/L GA_3_ can not (Chen et al., 2017). But the molecular mechanism of epicotyl dormancy in *P. cyrtonema* seeds remains unclear. In this study, we use transcriptomics and metabolomics to analyze the differences in *P. cyrtonema* seeds after exogenous 6-BA treatment. We identified the elongation of epicotyl buds as a critical period for dormancy release and identified the genes and metabolites related to epicotyl dormancy release, which provided a basis for the molecular regulation of epicotyl dormancy release and seedling emergence of *P. cyrtonema* seeds. We hope to make a preliminary exploration on the molecular mechanism of exogenous 6-BA relieving epicotyl dormancy of *P. cyrtonema* seeds through this study, so as to provide theoretical support for the promotion and development of Polygonatum industry.

## Materials and methods

### Plant materials and treatments

*Polygonatum cyrtonema* seeds in this study were collected in Sanyi Village, Chizhou, Anhui Province, China in September 2020 and identified by Professor Cai Yongping of Anhui Agricultural University. However, mature seeds were placed in wet sand at 4°C for 60 days. We remove the seeds from the sand and select the well-rounded seeds to sow in seedling trays with soil, then culture it in an artificial climate chamber (25°C, dark) and water it regularly. After 30 days, 200 mg/L 6-BA was sprayed on cormel with 0.1–0.2 cm bud length of epicotyl, and water was used as control. Seeds were collected at 0 day, 12, 24, 48, and 72 h, 7, 14, 21, 28, and 35 days. Collected seeds were cleaned surface impurities and quickly placed in liquid nitrogen for snap freezing, then stored in a −80°C freezer for further experiment analysis. The seed germination rate was counted every 5 days. The root length and bud length of *P. cyrtonema* seeds were measured with a ruler, and the emergence rate of *P. cyrtonema* seeds was counted by taking the first true leaf as the emergence. Three biological replicates per treatment, and 100 seeds per replicate.

### Determination of morphological indexes

Germination rate of *P. cyrtonema* seeds was counted every 5 days after sowing, with 100 seeds per replicate and three biological repetitions per period. Seed germination was regarded as the length of radicle breaking through the seed coat exceeding the length of the seed itself (Fu et al., 2017).

### Transcriptome sequencing

To identify the key genes regulating the differential stages, we performed high-throughput mRNA transcriptome sequencing on seeds of *P. cyrtonema* in three different stages. The seeds that grew into corms after sowing for 30 days were treated with 200 mg/L 6-BA, and the seeds at 12 h, 7 and 28 days after treatment were sampled (three biological replicates). The sampled seeds were frozen in liquid nitrogen and then preserved. RNA extraction from seeds by CTAB (QIAGEN, Germany), and a total of 21 RNA samples were processed using an Illumina NovaSeq 6000 (Illumina, United States) platform (Novogene, Beijing, China), which generated ∼4.2 Gb of sequencing data with 150-bp paired-end reads for each sample (Zhang Z. H. et al., 2019). A total of 225,180 unigenes were obtained and annotated in seven major databases. Data of RNA-Seq in this study have been deposited in the National Center for Biotechnology Information Gene Expression Omnibus (GEO) database (accession no: PRJNA833273).

### Identification and characterization of differentially expressed genes

The Illumina RNA-seq clean reads for 21 samples were separately mapped onto the above-mentioned transcript sequences using the Bowtie2 program in the RSEM software (Zhang Z. H. et al., 2019). The mapped read count of each transcript was calculated and further transformed into FPKM values, which were used as the expression levels in different samples. The DEGs between the two compared stages were determined using the DESeq R package (1.10.1), with the following criteria: | log2 (fold-change)| ≥ 1 and adjusted *p*-value < 0.05. The GO and KEGG enrichment analyses of the DEGs were performed using the GOseq R package (version 1.10.1) and the KOBAS software (version 2.0), respectively. The significance of the enriched GO terms and enriched KEGG pathways were separately assessed with the Wallenius non-central hypergeometric distribution test and the hypergeometric distribution test. An adjusted *p*-value < 0.05 was set as the threshold for significance (Liao et al., 2021).

### Quantitative real-time PCR assays

RNAprep pure plant kit (Biofit, Chengdu, China) was used to isolate total RNA from fresh sample (100 mg). According to the manufacturer’s instructions, RNA (1 μg) was used to synthesize cDNA using PrimeScript™ RT kit with gDNA eraser (January, Perfect Real Time, Takara, Tokyo, Japan). Using QuantStudio 6 Flex real-time PCR system (Thermo Fisher Scientific, Waltham, MA, United States) and SYBR^®^ Premix Ex Taq™ II (2x) (Japan, Takara), the gene expression level was detected by qRT-PCR. We used NCBI-BLAST online software to design fluorescent quantitative primers for the key genes in the zeatin and flavonoids biosynthesis pathways (Supplementary Table 1). The reaction steps are 50°C 2 min, 95°C 30 s, 95°C 5 s, 60°C 34 s, 40 cycles, and 72°C for 10 min. GAPDH were used as a reference gene to calculate the relative expression level of each target gene through the formula of 2^–ΔΔCT^, and the experiment was repeated three times (Li et al., 2021).

### Metabolomic analysis

For the metabolomics analysis, metabolic profiling of materials is to be sampled simultaneously with transcriptome (three biological replicates). The freeze-dried samples were crushed with a mixer mill for 60 s at 60 Hz. 50 mg aliquot of individual samples were precisely weighed and were transferred to an Eppendorf tube, after the addition of 700 μL of extract solution (methanol/water = 3:1, precooled at −40°C, containing internal standard). After 30 s vortex, the samples were homogenized at 35 Hz for 4 min and sonicated for 5 min in an ice-water bath. Repeats homogenize and sonicate for three times. Then the samples were extracted overnight at 4°C on a shaker. Then centrifuged at 12,000 rpm [RCF = 13800 (×g), *R* = 8.6 cm] for 15 min at 4°C. The supernatant was carefully filtered through a 0.22 μm microporous membrane, then the resulting supernatants were diluted five times with methanol/water mixture (v:v = 3:1, containing internal standard) and vortexed for 30 s and transferred to 2 mL glass vials, and take 30 μL from each sample and pooling as QC samples. Store at −80°C until the UHPLC-MS analysis. SCIEX Analyst Workstation Software (Version 1.6.3) was employed for MRM data acquisition and processing. MS raw data (.wiff) files were converted to the TXT format using MSconventer. In-house R program and database were applied to peak detection and annotation. The identification and structural analyses of the primary and secondary spectral data of the metabolites detected by mass spectrometry were based on the BIOTREE PWT database of Shanghai BIOTREE Biological Technology Co., Ltd. (Shanghai, China).

### Co-joint analysis of the transcriptome and metabolome

The DEGs and DEMs were mapped onto the KEGG pathways at the same stages. We performed correlation analysis on the screened DEGs and DEMs. The Corson program in R was used to calculate Pearson correlation coefficient. Networks were visualized by Cytoscape software v 3.9.1.

### Statistical analysis

Integrative analysis (such as significance, PCA analysis) of metabolome and transcriptome was carried out using R software (version 4.1.2, United States). Finally, visualization of Physiological Index Graph using GraphPad Prism (version 8, United States). Other graphics were visualized by Adobe Photoshop CC2021 (ADOBE, United States).

## Results

### Effects of 6-benzylaminopurine treatment on appearance and morphological indexes of *P. cyrtonema* seeds

**FIGURE 1:**
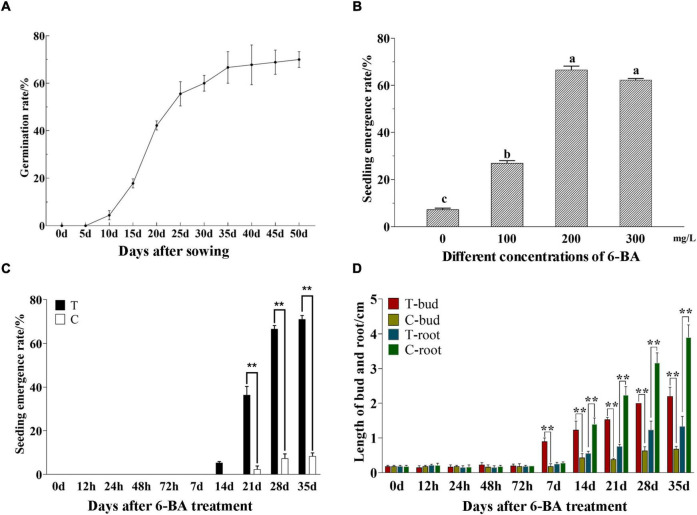
Determination of morphological indexes of seed germination and emergence of *P. cyrtonema*. **(A)** Germination rate of *P. cyrtonema* seeds at different times after sowing. **(B)** Effect of 6-BA treatment on seed germination rate of *P. cyrtonema*. Different letters indicated significant differences (*p* < 0.05) among different concentrations. **(C)** The emergence rate at different times after sowing. **(D)** The changes in bud length and root length of seeds after 200 mg/L 6-BA treatment. The concentration of 6-BA was 200 mg/L, and T represents the treatment group while C represents the control group in the same period. T-bud and T-root, respectively, represent the bud length and root length after 6-BA treatment; C-bud and C-root, respectively, represent the bud length and root length of the control group in the same period. “**” indicates that the difference is very significant.

The germination rate of *P. cyrtonema* seeds was increased with the extension of culture time and reached over 50% at 25 days after sowing. When the germination rate reached 60% (30 days), it was sluggish and had a tendency to be more consistent (Figure 1A). After that, the seeds stopped growing and entered epicotyl dormancy (Liao et al., 2021). This study found, to release the epicotyl dormancy of *P. cyrtonema* seeds, the application of a certain concentration of 6-BA could significantly increase the emergence rate then relieved the dormancy of seeds (Figure 1B). It can be seen that the application of 6-BA concentration at 100–300 mg/L can improve the emergence rate of *P. cyrtonema* seeds, but the emergence rate decreases when the concentration is higher than 200 mg/L.

Moreover, based on our previous experiments, seeds that sprouted out of cormel 30 days after planting were chosen and sprayed with 200 mg/L 6-BA to evaluate the physiological process of seed dormancy alleviation. The seeds were collected after treatment. In the meantime, water was applied as the control (CK). According to the emergence rate (Figure 1C), despite being treated for 7 days, the seeds failed to germinate. In the treatment group of 14 days the seeds successively emerged and the emergence rate reached over 60% after 28 days, while, in CK of the same period, a small number of seeds germinated from 21 days, but the emergence rate was always below 10%. Statistical results indicated that the bud length and root length (Figure 1D), there had no significant difference in the bud length of epicotyl within 72 h after treatment. From 7 days, the epicotyl buds in the treatment group obviously lengthened and continued to grow. But the buds in CK grew slowly. They did not exceed 1 cm in bud length when most of the seeds in the treatment group had already emerged, consistently maintaining a dormant state. At the same time, no significant differences in the root length could be observed within 7 days. From 14 days the root length in CK increased continuously, while it showed slow growth in the treated group. So, it can be seen that *P. cyrtonema* seeds exhibited a significantly higher emergence rate, faster bud growth, and were more susceptible to elongation and thus emergence. Unlike this, the growth of roots was relatively slowly.

### Metabolic profile of *P. cyrtonema* during seed germination

**FIGURE 2:**
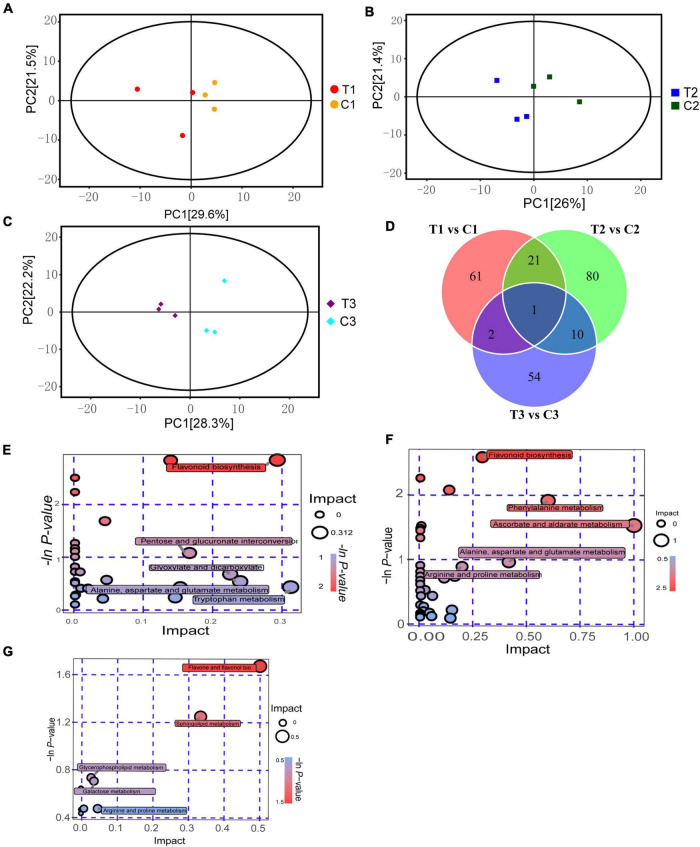
Metabolomics analysis on dormancy release of epicotyl in *P. cyrtonema* seeds. **(A)** PCA analysis of T1 vs. C1. **(B)** PCA analysis of T2 vs. C2. **(C)** PCA analysis of T3 vs. C3. **(D)** Venn diagram of metabolite distributions across different stages. **(E)** KEGG analysis of T1 vs. C1. **(F)** KEGG analysis of T2 vs. C2. **(G)** KEGG analysis of T3 vs. C3.

To understand the metabolic level differences in the process of epicotyl dormancy release in *P. cyrtonema* seeds, we selected seeds that grew into cormel 30 days after sowing (C0), and took materials, respectively, at the following stages: a short stage within 12 h after the application of exogenous 6-BA (T1), a stage that the elongation of epicotyl buds after 7 days (T2), seedling emergence stage after 28 days (T3), as well as the water controls in the same periods (C1, C2, and C3) (Supplementary Figure 1). Changes in 21 sample metabolites were identified by LC/MS/MS. The PCA analysis indicated that samples were all at the 95% confidence intervals, and intergroup differences in the samples were obvious (Figures 2A–C). A total of 1000 metabolites were detected in the samples, including 229 differential metabolites (DEMs). Among these identified DEMs, there were 61 DEMs that existed only in T1 vs. C1, mainly including 19 flavonoids, 7 alkaloids, and 6 phenolic, etc. 80 DEMs only existed in T2 vs. C2, which mainly included 24 flavonoids, 13 amino acids, and their derivatives and 4 carboxylic acids and their derivatives. There were 54 DEMs present only in T3 vs. C3, which mainly included 21 flavonoids, 4 alkaloids, and 3 aerobic compounds. In addition, Diosmin, a flavonoid, was the DEMs common to all stages (Figure 2D and Supplementary Table 2).

Orthogonal partial least squares-discriminant analysis (OPLS-DA) was used to study the identified metabolites. In the comparison combinations of T1 vs. C1, T2 vs. C2, and T3 vs. C3, based on VIP 1.0 together with *P*-value 0.05 as thresholds for significant differences, Q^2^Y were 0.78, 0.794, and 0.782, respectively. OPLS-DA scores showed very distinct results for the two groups, which all fell within the 95% confidence interval. We identified 85 DEMs (20 upregulated and 65 downregulated) in the comparisons between T1 and C1. Meanwhile, we identified 112 DEMs (30 upregulated and 82 downregulated) in the comparisons between T2 and C2. In addition, we identified 67 DEMs (31 upregulated and 36 downregulated) in the comparisons between T3 and C3 (Supplementary Figure 2).

We performed a KEGG functional annotation analysis for the DEMs in different stages and based on pathway analysis to further understand their biological functions. 25, 33, and 5 metabolic pathways were labeled in treated and control groups at 12 h, 7 and 28 days (Figures 2E–G). Enrichment analysis of DEMs revealed that a total of 38 DEMs were detected in T1 vs. C1, and significantly enriched pathways were flavonoid biosynthesis, nicotinate and nicotinamide metabolism, arginine and proline metabolism, and zeatin biosynthesis. While, in T2 vs. C2, a total of 60 DEMs were detected. Significantly enriched pathways were flavonoid biosynthesis, arginine and proline metabolism, and zeatin biosynthesis. In T3 vs. C3, a total of nine DEMs were detected. Significantly enriched pathways were flavone and flavonol biosynthesis, sphingolipid metabolism, and arginine and proline metabolism. It can be seen that the flavonoid biosynthetic pathway was significantly enriched in three stages, and a total of 37 metabolites were identified as flavonoids, seven of which were localized in the KEGG pathway, which were pelargonidin, naringenin, eriodictyol, naringenin chalcone, luteolin, kaempferol, and cyanidin. Meanwhile, arginine and proline were also the significantly enriched metabolism pathway in three stages, and a total of 37 metabolites were identified as arginine or proline, and four were localized in the KEGG pathway, which was citrulline, L-aspartic acid, hydroxyproline and L-glutamine, respectively. Moreover, zeatin biosynthesis was also significantly enriched in the first two stages (Supplementary Table 3), indicating that these metabolic pathways or metabolites were tightly related to dormancy release.

### Transcriptome analysis of *P. cyrtonema* seeds in different stages

**FIGURE 3:**
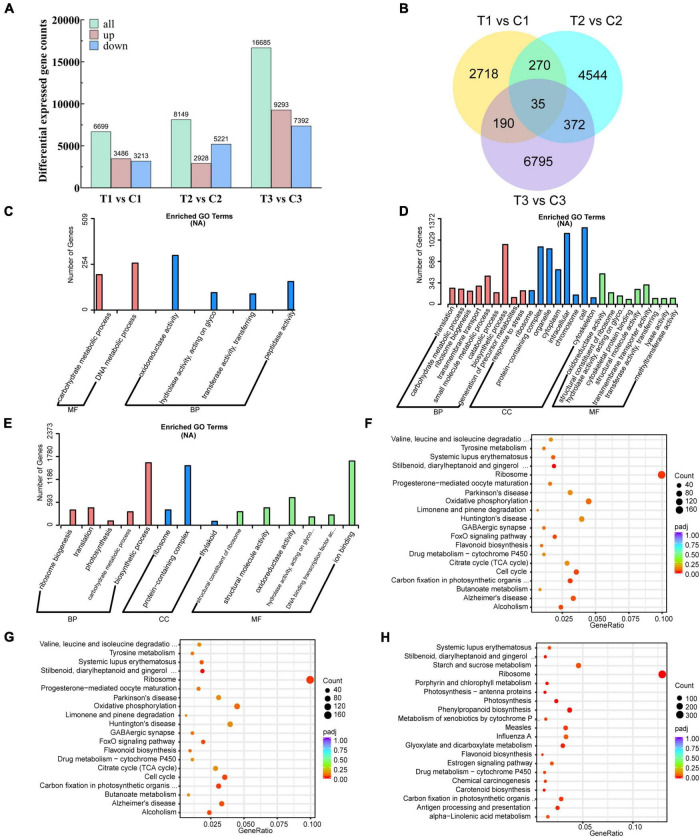
Transcriptomics analysis of *P. cyrtonema* seed at different stages. **(A)** Number of DEGs per comparison (both upregulated and downregulated). **(B)** Venn diagram showing the DEGs in the three comparison combinations. **(C)** GO annotation of DEGs of comparison group T1 vs. C1. **(D)** GO annotation of DEGs of comparison group T2 vs. C2. **(E)** GO annotation of DEGs of comparison group T3 vs. C3. **(F)** KEGG annotation of DEGs of comparison group T1 vs. C1. **(G)** KEGG annotation of DEGs of comparison group T2 vs. C2. **(H)** KEGG annotation of DEGs of comparison group T3 vs. C3.

To further investigate the potential regulatory molecular mechanisms of exogenous 6-BA relieving epicotyl dormancy of *P. cyrtonema* seeds, 21 cDNA libraries were constructed from the same samples used for metabolite data analysis and subjected to high-throughput RNA-seq analysis. With the comparison of three different stages, we obtained a total of 31,533 differentially expressed genes (DEGs). 3,486, 2,928, and 9,293 DEGs found to be the upregulated genes, while, 3,213, 5,221, and 7,392 DEGs belonged to the downregulated genes, respectively (Figure 3A). There were 2718, 4544, and 6795 DEGs that were differentially expressed only in the intergroup comparisons of T1 vs. C1, T2 vs. C2, and T3 vs. C3, respectively (Figure 3B).

In the comparison group at T1 vs. C1, we found a total of 11,586 DEGs, of which 4,590 were upregulated and 6,996 were downregulated (Supplementary Table 4). GO analysis of DEGs showed that biological processes affected included carbohydrate metabolic process. Molecular functions of DEGs associated with dormancy release included oxidoreductase activity, hydrolase activity, acting on glycosyl bonds, transferase activity, transferring glycosyl groups, and peptidase activity (Figure 3C and Supplementary Table 5). KEGG pathway analysis showed that the DEGs related to the dormancy release were mainly involved in stilbenoid, diarylheptanoid, and gingerol biosynthesis, zeatin biosynthesis, arginine, and proline metabolism, and flavonoid biosynthesis (Figure 3F and Supplementary Table 10).

In the comparison group at T2 vs. C2, we found a total of 18,058 DEGs, of which 7,127 were upregulated and 10,931 were downregulated (Supplementary Table 6). GO analysis of DEGs showed that biological processes affected included ribosome biogenesis, translation, photosynthesis, carbohydrate metabolic process and biosynthetic process. Molecular functions of DEGs associated with dormancy release included structural constituent of ribosome, structural molecule activity, oxidoreductase activity and hydrolase activity, acting on glycosyl bonds (Figure 3D and Supplementary Table 7). KEGG pathway analysis showed that the DEGs related to the dormancy release were mainly involved in stilbenoid, diarylheptanoid and gingerol biosynthesis, zeatin biosynthesis, flavonoid biosynthesis, arginine and proline metabolism (Figure 3G and Supplementary Table 11).

In the comparison group at T3 vs. C3, we found a total of 29,132 DEGs, of which 14,421 were upregulated and 14,711 were downregulated (Supplementary Table 8). GO analysis of DEGs showed that biological processes affected included ribosome biogenesis, translation, photosynthesis, carbohydrate metabolic process, and biosynthetic process. Molecular functions of DEGs associated with dormancy release included structural constituent of ribosome, structural molecule activity, oxidoreductase activity, hydrolase activity, acting on glycosyl bonds, and DNA binding transcription factor activity (Figure 3E and Supplementary Table 9). KEGG pathway analysis showed that the DEGs related to the dormancy release were mainly involved in photosynthesis, carotenoid biosynthesis, flavonoid biosynthesis, zeatin biosynthesis, and arginine and proline metabolism (Figure 3H and Supplementary Table 12).

### Changes of genes and metabolites in flavonoid biosynthesis, arginine and proline metabolism, and zeatin biosynthetic pathways during epicotyl dormancy release of *P. cyrtonema* seeds

**FIGURE 4:**
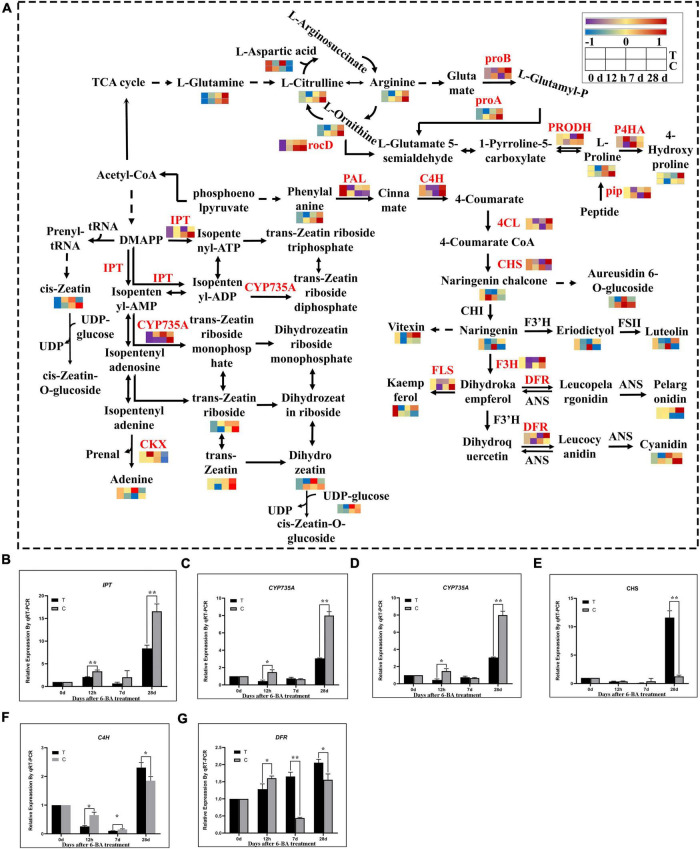
Integrated transcriptomics and metabolomics analysis of flavonoid biosynthesis, arginine and proline metabolism, and zeatin biosynthetic pathways. **(A)** The DEGs and DEMs involved in the flavonoid biosynthesis, arginine and proline metabolism, and zeatin biosynthetic pathway at different stages. The color gradient, ranging from blue, through yellow, to red represents low, middle and high FPKM of genes. The color gradient, ranging from purple, through yellow, to red represents low, middle and high relative abundance of metabolites. T, Treatment group; C, Control group. **(B–G)** Verification of related genes in transcriptome by qRT-PCR. *IPT*, isopentenyl transferases gene; *CYP735A*, cytokinin hydroxylase gene; *CKX*, cytokinin degrading enzyme gene; *CHS*, chalcone synthase gene; *C4H*, cinnamate-4-hydroxylase; *DFR*, dihydroflavonol 4-reductase; “*” indicates that the difference is significant, “**” indicates that the difference is very significant.

In the analysis of transcriptome and metabolome, we found that the flavonoid biosynthesis, arginine and proline metabolism, and zeatin biosynthetic pathway was significantly enriched in all stages. Therefore, we further analyzed the expression patterns of genes and metabolites of the these three pathway (Figure 4). Compared with CK of the same period, at 12 h most genes involved in the flavonoid biosynthetic pathways including *PAL, CYP73A, 4CL*, and *F3’H*, were significantly downregulated, and metabolites such as pelargonidin, kaempferol, luteolin, naringenin, and eriodictyol were also significantly decreased, indicating that most of genes and metabolites of the flavonoid biosynthetic pathway were significantly decreased at this time. But at 7 days, all genes except *PAL* and *CYP73A* showed a significant decrease in expression, and significant decreases were observed in all metabolites except naringenin chalcone, cyanidin, kaempferol, and vitexin, which means that most genes and metabolites of the flavonoid biosynthetic pathway were significantly decreased at this time. At 28 days, except for *F3’H* and *FLS*, all other genes were significantly upregulated. All metabolites except naringenin chalcone, aureusidin 6-O-glucoside and cyanidin rose significantly, indicating that most genes and metabolites of the flavonoid biosynthetic pathway rose significantly at this time.

Further analysis showed that the metabolites involved in arginine metabolism, including L-arginine, continued to increase in three stages, but were significantly lower than the CK of the same period, except L-glutamine and L-aspartic acid. Studies have shown that the arginine in coniferous pines in overwintering dormancy accumulates significantly while glutamine maintains low levels (Durzan, 2009). In this study arginine content during dormancy relief of *P. cyrtonema* seeds was low in the treatment group while glutamine was maintained at a high level throughout. This illustrates from the side that *P. cyrtonema* seeds could use arginine to accelerate amino metabolism, thereby promoting the relief of dormancy. While in the proline metabolism, compared with the CK of the same period genes and metabolites involved in proline biosynthesis metabolic showed a tendency to increase first but then decrease in the three stages of the treatment group, and were more abundant at 7 days. Whereas the proline content in CK was consistently elevated and was higher than that in the treated group at 28 days. At 7 days, the proline content in the treated group was higher than that in the CK. In contrast to the proline synthesis pathway, 4-hydroxyproline, a catabolite of proline, showed a tendency to decrease first and then increase in the three stages in the treated group and was lower than the control group at 7 days. While *P4HA*, a proline catabolic gene, showed the same trend with metabolites. Proline had a significant rise in both its biosynthetic gene and metabolites at 7 days, but its catabolic genes and metabolites had a significant decline at 7 days. Proline plays an important role in the release of plant dormancy. Research shows that proline had an important role in the process of grape bud dormancy release (Mujahid et al., 2020). Therefore, proline was found to be elevated at 7 days during the epicotyl dormancy release of *P. cyrtonema* seeds and may have promoted this process. At 28 days, the proline content was lower than in the CK of the same period, which is similar to that at 12 h, indicating that the elongation of the epicotyl bud of *P. cyrtonema* seeds may be the critical period for dormancy release.

Zeatin was the first plant natural cytokinin to be discovered, and we analyzed the expression patterns of genes and metabolites of the zeatin biosynthetic pathway. Compared with the CK of the same period the genes involved in zeatin biosynthesis, *IPT (adenosine phosphates isopentenyltransferase)* and *CYP735A (cytokinin trans hydroxylase)*, as well as their metabolites, were significantly upregulated at 12 h and 7 days, and downregulated at 28 days. And cytokinin dehydrogenase (CKX) is a negative regulatory enzyme of zeatin in zeatin metabolism, which regulates the content of zeatin by degrading the metabolic product of the first step of zeatin synthesis. It can be seen that at 12 h, the zeatin and adenine content increased, the metabolite of zeatin catabolism was also increased. At 7 days, there was a general increase in zeatin content and a decrease in *CKX* expression. At 28 days, zeatin content was generally reduced while *CKX* expression began to rise. Overall, during the first 7 days genes and metabolites involved in zeatin synthesis pathway were generally increased, and after 28 days, the associated genes and metabolites had a general decrease. Being exactly opposite to the synthetic pathway, the genes involved in zeatin metabolism predicted the essential role of cytokinin dehydrogenases as negative regulatory enzymes of zeatin in the regulation of cytokinin levels.

To verify the credibility of the transcriptome information, six genes related to zeatin and flavonoid biosynthesis were selected for validation by qRT-PCR. The qRT-PCR results showed that the expression of these genes were very similar to the RNA-seq results (Figures 4B–G).

### Changes of genes and metabolites in plant hormone pathway during epicotyl dormancy release of *P. cyrtonema* seeds

Phytohormones played an important role in the process of plant dormancy release, of which comparatively important are indole acetic acid (IAA), gibberellic acid (GA), abscisic acid (ABA), and cytokinin (CTK) (Ito et al., 2021). In the result of 2.4, we have analyzed the expression patterns of genes related to the zeatin biosynthetic pathway of CTK. And then we counted DEGs in plant hormone pathway, and finally enriched them in GA, ABA and IAA metabolic pathways.

**FIGURE 5:**
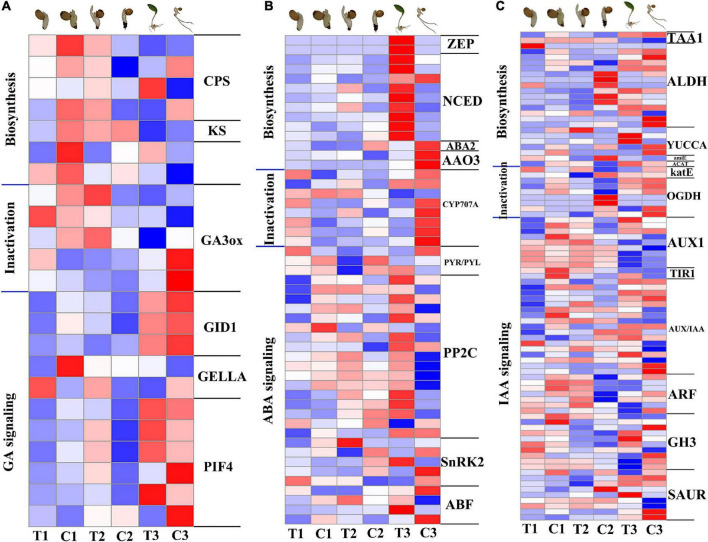
Expression patterns of genes related to GA, ABA, and IAA biosynthesis, inactivation and signal transduction pathways in transcriptome. **(A)** Heat map of gene expression related to GA pathway. **(B)** Heat map of gene expression related to ABA pathway. **(C)** Heat map of gene expression related to IAA pathway. The color gradient, ranging from blue, through white, to red represents low, middle and high expression of genes. Log_2_(FC) ranges from –1 to 1.

GA pathways, including GA biosynthesis, metabolism and signal transduction (Figure 5A), were all significantly downregulated at 12 h compared with the CK in the same period. The genes *GIBBERELLIN INSENSITIVE DWARF1* (*GID1*), *DELLA protein* (*DELLA*), and *phytochrome-interacting factor 4* (*PIF4*) of GA signal transduction pathway were also significantly downregulated. At 7 days, *PIF4* as well as *gibberellin 2-beta-dioxygenase* (*GA2ox*), which is part of the GA catabolic pathway, were significantly upregulated. At 28 days, *gibberellin 3-beta-dioxygenase* (*GA3ox*) was upregulated and *DELLA* was downregulated, indicating the promotion of GA pathway on dormancy release. ABA pathways, including ABA biosynthesis, metabolism and signal transduction (Figure 5B), compared with the CK in the same period several genes involved in ABA biosynthesis and signal transduction were upregulated in the three stages, reflecting the importance of the ABA signaling pathway for dormancy release and emergence from *P. cyrtonema* seeds. Genes related to ABA degradation including *CYP707A (abscisic acid 8’-hydroxylase)* were upregulated in the first 7 days and significantly downregulated at 28 days. IAA pathways, including IAA biosynthesis, metabolism and signal transduction (Figure 5C), many biosynthetic and signal transduction genes were significantly downregulated at 12 h compared with the CK of the same period. YUCs function as flavin-containing monooxygenases (FMO) catalyzing the rate-limiting irreversible oxidative decarboxylation of indole-3-pyruvate acid (IPyA) to form indole-3-acetic acid (IAA) (Cao et al., 2019). However, *YUCCA* in the transcriptome rose significantly at 7 days, indicating a regulatory role of the IAA pathway in this process.

### Association analysis of flavonoid biosynthesis, arginine and proline metabolism, and zeatin biosynthetic pathways

**FIGURE 6:**
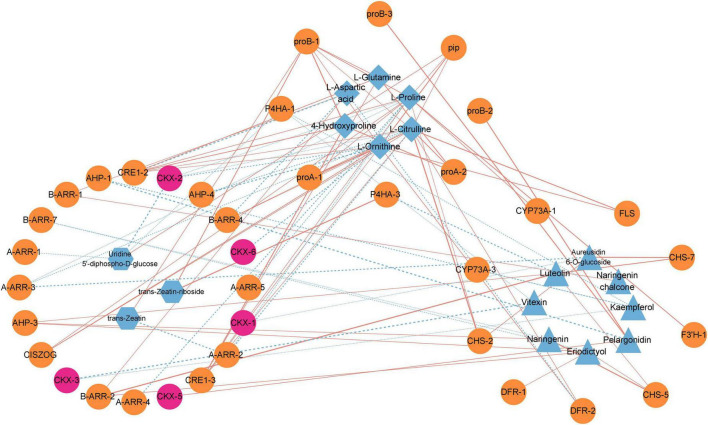
Correlation analysis of flavonoid biosynthesis, arginine and proline metabolism, and zeatin biosynthesis pathways. Orange blocks represent genes, blue blocks represent metabolites, and pink blocks represent *PcCKXs*. Red line represents positive correlation and blue line represents negative correlation.

Our previous results illustrated that it might be the critical period for dormancy release in the elongation of epicotyl buds of *P. cyrtonema* seeds, in which genes and metabolites of flavonoid biosynthesis, arginine and proline metabolism, and zeatin biosynthesis pathways play important roles. Therefore, we performed an association analysis between DEGs and DEMs in three pathways in T2 vs. C2 (Figure 6). We could found that L-citrulline, L-glutamine and L-ornithine in the arginine and proline metabolism pathway were significantly correlated with most genes. Furthermore, we noticed that in the zeatin biosynthetic pathway genes involved in cytokinin dehydrogenases *(PcCKXs)* of *P. cyrtonema* were active in three stages, which showed significant positive correlations with pelargonidin in the flavonoid pathway and negative correlations with L-proline, L-ornithine, and L-citrulline in arginine and proline metabolism. Previous studies illustrated that genes and metabolites of the flavonoid, arginine and proline metabolic pathways were significantly under-expressed during the first 7 days, meaning that they could negatively regulate dormancy release. It means that *PcCKXs* are significantly associated with them. This suggests that *PcCKXs* were closely related to flavonoid pathway, arginine and proline metabolism pathway, and affected the dormancy release *P. cyrtonema* seeds.

## Discussion

Seed dormancy is the result of natural evolution of plants in response to adverse environments, but it is not conducive to agricultural production for human beings (Agrawal et al., 2021). Seed dormancy is regulated by temperature, light, hormones and other factors, and affects mRNA splicing, translation and chromatin remodeling (Chen D. L. et al., 2020; Tognacca and Botto, 2021). *P. cyrtonema* seeds began to enter the epicotyl dormancy after the bulb appeared. The dormancy state can be broken effectively by alternating temperatures (Liao et al., 2021). However, other studies have shown that exogenous phytohormones can also relieve dormancy (Yang et al., 2017). Interestingly, many studies have reported that exogenous GA_3_ can relieve seed dormancy (Ghannad et al., 2022), but it can not relieve epicotyl dormancy of *P. cyrtonema* seeds (Chen et al., 2017). In this study, morphological observation showed that the seeding emergence rate of *P. cyrtonema* seeds after 30 days of sowing was significantly improved after exogenous 6-BA hormonal treatment. When the concentration of 6-BA was 100–300 mg/L, the effect was obvious, and the optimum concentration was 200 mg/L. This indicated that exogenous 6-BA could relieve the epicotyl dormancy of *P. cyrtonema* seeds. At the same time, the roots gradually elongated to complete the uptake of external nutrients. But from the same period of the water control group, after entering the epicotyl dormancy, the epicotyl bud elongation was not obvious, but the root was growing rapidly and was longer than the treatment group after 14 days.

Metabolomics and transcriptomics were used to analyze the epicotyl dormancy release process of *P. cyrtonema* seeds. PCA and Venn diagram analysis showed that there were significant differences between the treatment group and the control group, among which flavonoid substances were significantly different. GO analysis showed that the activities of oxidoreductase, hydrolase, mRNA processing and peptidase were active during dormancy release. Research shows that storage proteins are degraded to provide nutrients and energy required, inter alia, for the translation of stored mRNA and biosynthesis of new proteins with defined function in the imbibition phase of seed germination. Proteasome activity is required for germination, which plays central roles in cellular amino acids homeostasis. In the DEG part they only mentioned peptidase (Liu et al., 2019). In the KEGG pathway enrichment analyses of transcriptome and metabolome, we also found that the flavonoid biosynthetic pathway was always enriched in each period, indicating that it might be play an important role in the epicotyl dormancy release of *P. cyrtonema* seeds. From the differential expression profiles of genes and metabolites in flavonoid biosynthetic pathway, it could be seen that within 7 days after dormancy release, exogenous 6-BA treatment made most of them lower than those in the control group at the same time. Studies have shown that flavonoids play a regulatory role in flower buds differentiation, which is embodied in the decrease of flavonoids contents during flower buds differentiation (Ma and Zu, 2009). Therefore, flavonoids have a certain inhibitory effect on flower bud differentiation of plants. In addition, flavonoids represented by quercetin and kaempferol which were significantly increased in dormant buds of sweet cherry (Michailidis et al., 2018). This indicated that 6-BA treatment inhibited the synthesis of flavonoids, thereby relieving the inhibitory effect on epicotyl bud differentiation of *P. cyrtonema* seeds, and the seeds could complete bud differentiation. At 28 days, 6-BA treatment made the seeds grow into seedlings, while the control group took root without emergence. Most studies have shown that flavonoids are auxin transport inhibitors (Brown et al., 2001),and auxin has the function of accelerating cell elongation to participate in root growth regulation. Therefore, at 28 days, the genes and metabolites of the flavonoid biosynthetic pathway in the treatment group increased, which inhibited the transportation of auxin and made the seeds more seeding rather than rooted. In the transcriptome analysis, we found that auxin synthesis gene *YUCCA* and signal transduction gene *ARF* were significantly downregulated, indicating that the increase of flavonoid content after 6-BA treatment inhibited the expression of auxin synthesis genes. In summary, in the process of 6-BA treatment to relieve epicotyl dormancy of *P. cyrtonema* seeds, the inhibition on bud differentiation was relieved by inhibiting the genes and metabolites of flavonoid biosynthesis pathway in the first 7 days, and the expression of auxin-related genes were promoted, thereby promoting the elongation of epicotyl buds. In the later stages, the expression of auxin synthesis genes was inhibited by promoting flavonoid related genes and metabolites, which further inhibited root growth and promoted the transformation of seeds to seeding emergence. But there are also reports that flavonoids accumulate during seed germination (Liu R. et al., 2021). This indicated that the molecular mechanisms of epicotyl dormancy release and germination of *P. cyrtonema* seed were different.

Arginine and proline play important roles in plant dormancy release. We note that most of the genes and metabolites involved in arginine biosynthesis were downregulated in three stages. Research shows that ornithine and L-asparagine showed consumption patterns during rice germination according to our results (Guo et al., 2021). This indicates that arginine was always used as a donor to provide proline during the dormancy release of epicotyl of *P. cyrtonema* seeds. Proline contents were significantly upregulated at 7 days, and downregulated at 12 h and 28 days. Research shows that in the treatment of dormant grape bud, when proline accumulates, it protects the cells against oxidative stress and upregulates the oxidative pentose phosphate pathway causing a series of events that lead to the release of dormancy (Mujahid et al., 2020), this suggests that proline promotes dormancy release. Similarly, free proline content in roots of alfalfa with low fall dormancy was significantly higher than that of alfalfa with high fall dormancy (Liu et al., 2007). This further indicates that proline plays an important role in plant dormancy release. Therefore, the genes and metabolites in the proline metabolic pathway were significantly upregulated at 7 days, which was an important period for promoting dormancy release, because proline had a positive regulatory effect on dormancy release. The content of proline decreased in the early and late stages, which may be that proline did not maintain a high level to promote bud elongation in these two stages, so the content of proline was lower than that of the control group. Therefore, we believe that proline significantly increased in the elongation of epicotyl buds, the epicotyl dormancy release plays an important role.

Furthermore, we discovered that plant hormones play a crucial role in dormancy release. During seed dormancy, cytokinin antagonizes ABA to induce dormancy release (Huang et al., 2018). Seed dormancy is mainly controlled by abscisic acid ABA and GA, and ABA decreased and GA increased during seed dormancy release in Arabidopsis (Chen H. et al., 2020). However, *NCED* of ABA biosynthetic pathway were upregulated in the three stages of treatment groups, and during the development of *P. cyrtonema* seed from corm to seedling emergence, the development of embryo needed to transport nutrients from endosperm to new corm tissue and cold stratification before seedling establishment (Liao et al., 2021). This means that seeds are still not fully mature, and there is a direct correlation between ABA excess production and fruit maturity (Monribot-Villanueva et al., 2022). So the genes of the ABA biosynthetic pathway always upregulated in three stages may be to complete the seed maturation. However, in ABA catabolism, *CYP707A* was significantly upregulated at 12 h and 7 days and downregulated at 28 days in the treatment group. ABA catabolism plays an important role in the regulation of active hormone levels during seed development. In Arabidopsis *cyp707a* mutant, enhanced dormancy and ABA accumulation has further proven the important contribution of CYP707A1 and CYP707A2, which transcripts are highly accumulated at early and late maturation stages, respectively (Sano and Marion-Poll, 2021). In cereals, ABA catabolism by CYP707A family members has also been described to regulate ABA levels in developing seeds (Tuan et al., 2018). In addition, when *CYP707A* was overexpressed, the dormancy release of pear is accelerated (Li et al., 2018), which means that *CYP707A* plays an important role in regulating ABA homeostasis and relieving plant dormancy. GA pathway can indirectly regulate seed dormancy solution (Cao et al., 2020), and the *CPS, KS, GA3ox* of GA biosynthesis pathway genes were downregulated in the 12 h treatment group, which may be due to the antagonism of GA to ABA (Ye and Zhang, 2012). But with the release of epicotyl dormancy, GA-related genes began to be upregulated, promoting bud elongation. The change in IAA pathway was similar to that of GA pathway. The key enzyme gene *YUCCA* of IAA biosynthesis pathway which was upregulated at 7 days. Research showed that low concentration of 1-naphthaleneacetic acid (NAA) or 2,4-dichlorophenoxyacetic acid (2,4-D) could promote seed germination assayed by cotyledon greening (He et al., 2012). This was consistent with our study, which further indicated the key role of the elongation of epicotyl buds in the dormancy release of *P. cyrtonema* seeds. It was worth noting that the zeatin biosynthetic pathway belonging to cytokinin also played an important role in the epicotyl dormancy release of *P. cyrtonema* seeds. IPT is a rate-limiting enzyme for zeatin biosynthesis pathway (Frébort et al., 2011; Chen K. et al., 2020). Cytokinin trans-hydroxylase (CYP735A) catalyzes the production of the previous step to form trans-zeatin, which is one of the main active forms of cytokinin in plants.

In this study, at 12 h and 7 days after exogenous 6-BA treatment, the gene expression levels of *IPT* and *CYP735A* were significantly lower than those in the control group at the same time. However, in the analysis of metabolic data, it was observed that the contents of major cytokinin such as trans-zeatin and trans-zeatin riboside were significantly higher than those in the control group. From the perspective of cytokinin catabolism, cytokinin degrading enzyme gene *CKX* increased significantly at 12 h and decreased significantly at 7 days. We can speculate that *CKX* gene was significantly downregulated at 7 days, which reduced the degradation of cytokinin in the treatment group, and thus the content of cytokinin was higher than that in the control group. Exogenous cytokinin increased endogenous cytokinin content in *P. cyrtonema* seeds in a short time. Therefore, the expression of cytokinin biosynthesis genes *IPT* and *CYP735A* decreased, while the expression of cytokinin degradation pathway gene *CKX* increased. This indicates that in the early stage of dormancy release of *P. cyrtonema* seeds, especially on the 7 days, it inhibited the degradation of cytokinin by reducing the expression of *CKX*, so that cytokinin was higher in the treatment group. The genes of *CKX* played an important role in cytokinin catabolism. Studies have shown that specific expression of *CKX* in maize roots leads to greater root formation (Ramireddy et al., 2021). In potatoes, the tubers overexpressing *CKX* showed a long dormancy period, indicating the negative regulatory role of *CKX* in dormancy release (Hartmann et al., 2011). In the transcriptome, the expression of *PcCKXs* were significantly downregulated at 7 days, which further confirmed our previous hypothesis that the elongation of epicotyl buds is a critical period for dormancy release. From the results of correlation analysis, *PcCKXs* were significantly positively correlated with flavonoid biosynthesis pathway, while negatively correlation with arginine and proline metabolism pathway, indicating that *PcCKXs* has a negative regulatory role in the process of dormancy release of *P. cyrtonema*.

**FIGURE 7:**
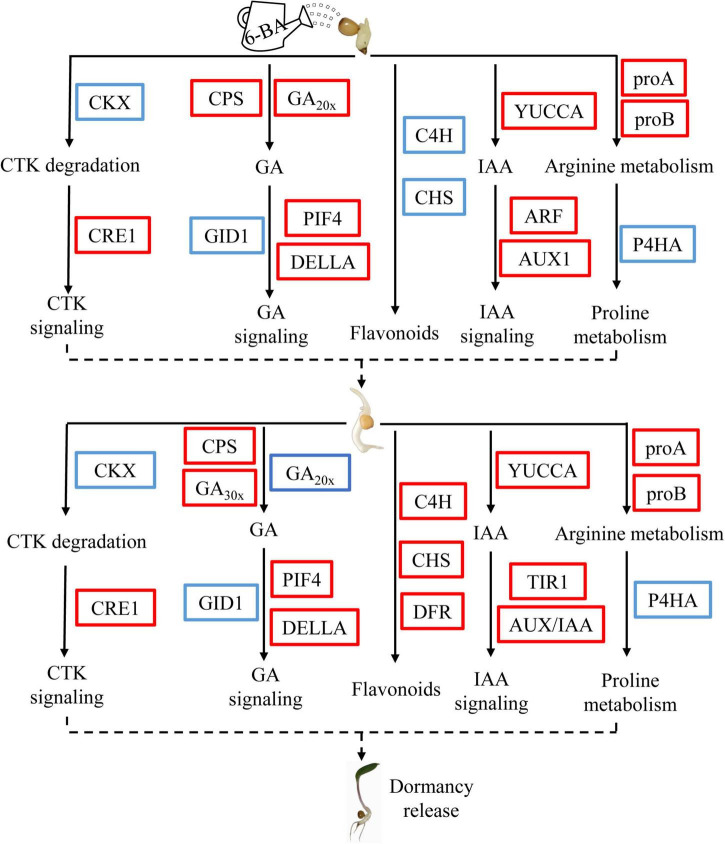
Schematic model showing the changes of a metabolic pathway in the critical period of epicotyl dormancy release of *P. cyrtonema* seeds. The red box and blue box indicate upregulated and downregulated genes in this pathway, respectively.

In summary, we determined the elongation of epicotyl buds as a critical period for dormancy release of *P. cyrtonema* seeds by applying exogenous 6-BA, and preliminarily explained the related mechanisms through transcriptome and metabolomics (flavonoid biosynthesis, arginine and proline metabolism, zeatin biosynthesis) (Figure 7). In this model, exogenous 6-BA treatment promotes GA biosynthesis and signal transduction pathway, and induce the release of epicotyl dormancy of *P. cyrtonema* seeds. At the same time, exogenous 6-BA treatment may increase the content of endogenous cytokinin in the elongation of epicotyl buds by inhibiting the expression of cytokinin dehydrogenase gene *(PcCKXs)*. In addition, exogenous 6-BA treatment may increase auxin content and decrease flavonoid content, and ultimately induce the release of epicotyl dormancy of *P. cyrtonema* seeds. Otherwise, exogenous 6-BA treatment promotes decomposition of arginine, and induces proline metabolism and reduces proline metabolism gene expression, and further induces the release of epicotyl dormancy of *P. cyrtonema* seeds. At the seed emergence stage (28 days), the change trend of these pathways was basically the same as that of 7 days, but the genes of flavonoid synthesis pathway were different from those in the earlier stage, and their gene expression was significantly upregulated. Future research will focus on the functional role of these key factors in the dormancy release of *P. cyrtonema* seeds.

## Conclusion

In this study, we explored the molecular mechanism of exogenous 6-BA treatment on the dormancy release of epicotyl of *P. cyrtonema* seeds. The results showed that exogenous 6-BA treatment could significantly promote the shoot length of epicotyl and increase the emergence rate of *P. cyrtonema* seeds. During the dormancy release of epicotyl of *P. cyrtonema* seeds, the contents of trans-zeatin, proline, auxin and gibberellin was higher, while the contents of flavonoids and arginine were low in the treatment group. Cytokinin dehydrogenase gene *PcCKXs* has a key correlation with pelargonidin in flavonoid biosynthesis and L-proline, L-ornithine and L-citrulline in arginine and proline metabolism, these results provided a new insight into the molecular mechanism of exogenous 6-BA relieving epicotyl dormancy of *P. cyrtonema* seeds.

## Data availability statement

Data of RNA-Seq in this study have been deposited in the National Center for Biotechnology Information Gene Expression Omnibus (GEO) database (accession no: PRJNA833273).

## Author contributions

YC and QJ participated in research design. WZ and LX conducted the experiments. WZ, LX, and FP performed the data analysis. WZ, MM, and CS wrote or contributed to the writing of the manuscript. All authors contributed to the article and approved the submitted version.

## Supplementary material

Supplementary Figure 1 Seeds of *P. cyrtonema* at various stages of transcriptome collection.

Supplementary Figure 2 Score plot of the OPLS-DA of pairwise comparisons.

Supplementary Table 1 Primers used for qRT-PCR analysis.

Supplementary Table 2 KEGG Enrichment Analysis of DEMs of *P. cyrtonema* Seeds in Different stages.

Supplementary Table 3 Metabolites of *P. cyrtonema* seeds in different stages.

Supplementary Table 4 Statistics of differentially expressed genes in T1 vs. C1.

Supplementary Table 5 GO analysis of differentially expressed genes in T1 vs. C1.

Supplementary Table 6 Statistics of differentially expressed genes in T2 vs. C2.

Supplementary Table 7 GO analysis of differentially expressed genes in T2 vs. C2.

Supplementary Table 8 Statistics of differentially expressed genes in T3 vs. C3.

Supplementary Table 9 GO analysis of differentially expressed genes in T3 vs. C3.

Supplementary Table 10 KEGG analysis of differentially expressed genes in T1 vs. C1.

Supplementary Table 11 KEGG analysis of differentially expressed genes in T2 vs. C2.

Supplementary Table 12 KEGG analysis of differentially expressed genes in T3 vs. C3.

## Abbreviations

6-BA: 6-benzylaminopurine
PCA: principal component analysis
OPLS-DA: orthogonal partial least squares-discriminant analysis
DEGs: differentially expressed genes
DEMs: differentially expressed metabolites
GA: gibberellic acid
ABA: abscisic acid
IAA: 3-indoleacetic acid.
