# Supplementary figures and images for: Transcriptomics and metabolomics changes triggered by exogenous 6-benzylaminopurine in relieving epicotyl dormancy of Polygonatum cyrtonema Hua seeds
Source: Front Plant Sci. 2022 Jul 25;13:961899. doi: 10.3389/fpls.2022.961899 (PMC9358440; doi:10.3389/fpls.2022.961899)

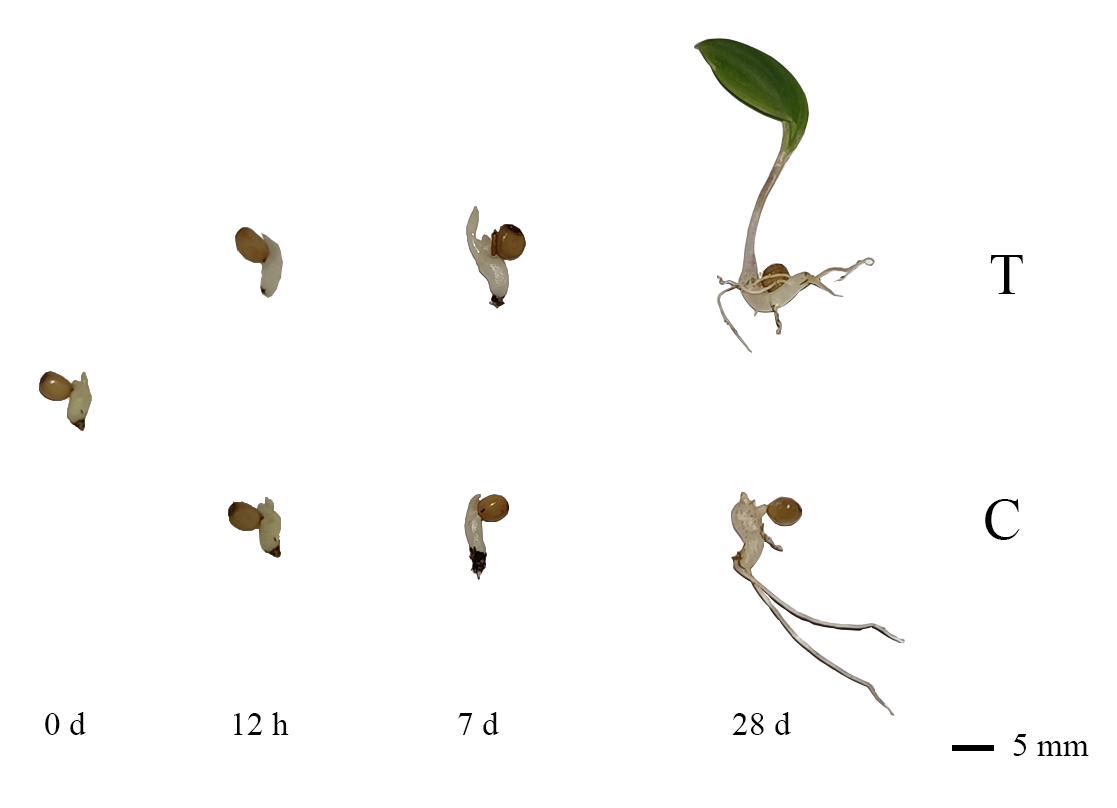

Supplement: Supplementary Figure 1 — Seeds of P. cyrtonema at various stages of transcriptome collection. [file Image_1.TIF]

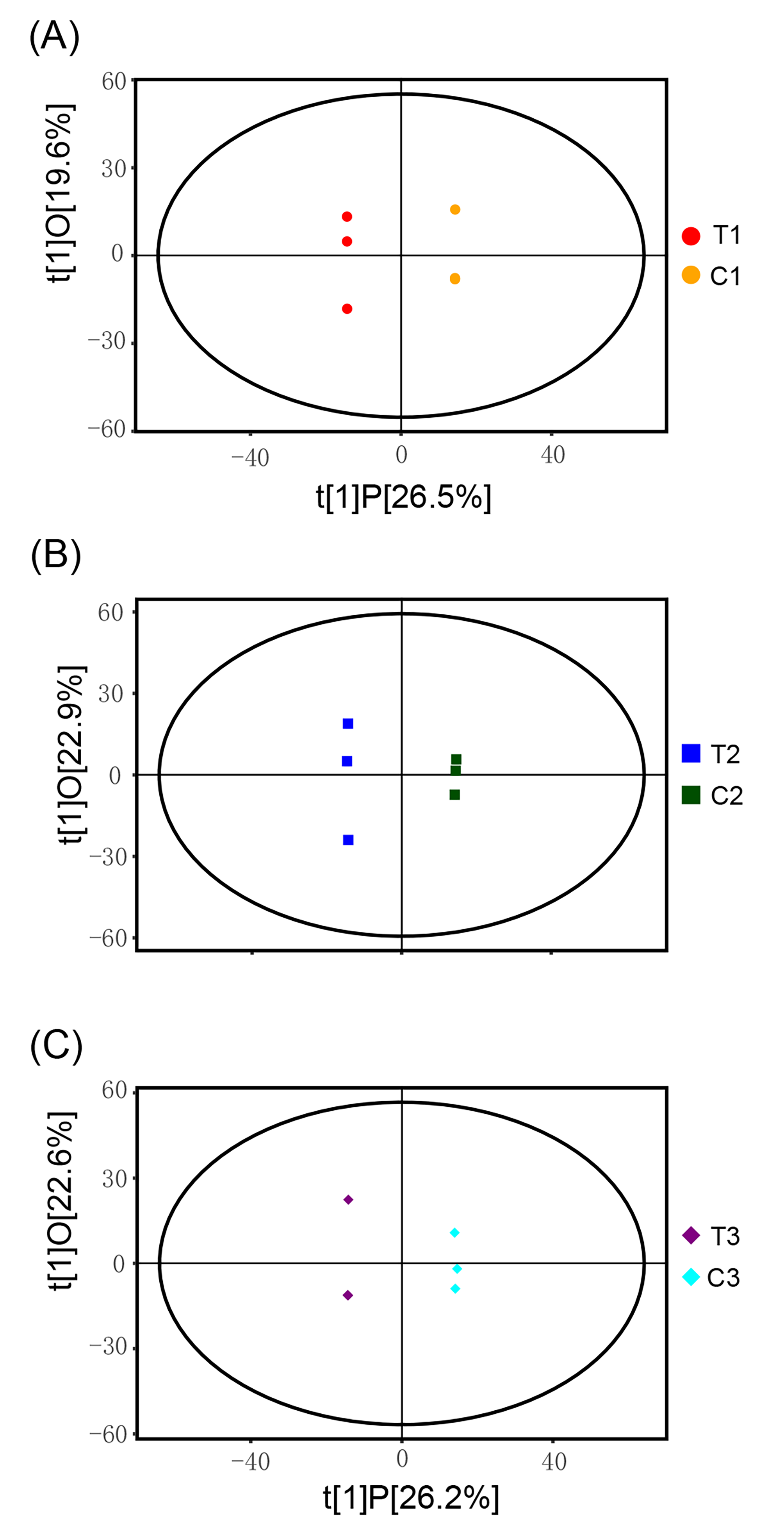

Supplement: Supplementary Figure 2 — Score plot of the OPLS-DA of pairwise comparisons. [file Image_2.TIF]
