# Supplementary material for: Transcriptomics and metabolomics changes triggered by exogenous 6-benzylaminopurine in relieving epicotyl dormancy of Polygonatum cyrtonema Hua seeds
Source: Front Plant Sci. 2022 Jul 25;13:961899. doi: 10.3389/fpls.2022.961899 (PMC9358440; doi:10.3389/fpls.2022.961899)
Supplement: Supplementary Table 1 — Primers used for qRT-PCR analysis. [file Table_1.docx]

Supplementary Table 2 KEGG Enrichment Analysis of DEMs of *P.cyrtonema* Seeds in Different stages

| **Pathway** | **Hits** | **Raw p** | **-ln(p)** | **Hits Cpd** |
| --- | --- | --- | --- | --- |
| **T1 vs. C1** |  |  |  |  |
| Flavonoid biosynthesis | 4 | 0.058246 | 2.8431 | Cyanidin cpd:C05905;  Naringenin cpd:C00509;  Eriodictyol cpd:C05631;  Naringenin chalcone cpd:C06561 |
| Nicotinate and nicotinamide metabolism | 2 | 0.058607 | 2.8369 | L-Aspartic acid cpd:C00049;  Nicotinamide ribotide cpd:C00455 |
| beta-Alanine metabolism | 2 | 0.081673 | 2.505 | L-Aspartic acid cpd:C00049;  Uracil cpd:C00106 |
| Glycerolipid metabolism | 2 | 0.10703 | 2.2346 | Uridine diphosphate glucose cpd:C00029;  Glyceric acid cpd:C00258 |
| Arginine and proline metabolism | 3 | 0.18538 | 1.6853 | Citrulline cpd:C00327;  L-Aspartic acid cpd:C00049;  Hydroxyproline cpd:C01157 |
| Glucosinolate biosynthesis | 1 | 0.2821 | 1.2655 | L-Tryptophan cpd:C00078 |
| Lysine biosynthesis | 1 | 0.31135 | 1.1668 | L-Aspartic acid cpd:C00049 |
| Glycine, serine and threonine metabolism | 2 | 0.32958 | 1.1099 | L-Aspartic acid cpd:C00049;  L-Tryptophan cpd:C00078 |
| Pentose and glucuronate interconversions | 1 | 0.33944 | 1.0805 | Uridine diphosphate glucose cpd:C00029 |
| Cyanoamino acid metabolism | 1 | 0.3664 | 1.004 | L-Aspartic acid cpd:C00049 |
| Ascorbate and aldarate metabolism | 1 | 0.44099 | 0.81874 | Uridine diphosphate glucose cpd:C00029 |
| Pantothenate and CoA biosynthesis | 1 | 0.48586 | 0.72183 | Uracil cpd:C00106 |
| Zeatin biosynthesis | 1 | 0.48586 | 0.72183 | Uridine diphosphate glucose cpd:C00029 |
| Glyoxylate and dicarboxylate metabolism | 1 | 0.50696 | 0.67932 | cis-Aconitic acid cpd:C00417 |
| Citrate cycle (TCA cycle) | 1 | 0.5653 | 0.5704 | cis-Aconitic acid cpd:C00417 |
| Carbon fixation in photosynthetic organisms | 1 | 0.5832 | 0.53922 | L-Aspartic acid cpd:C00049 |
| Alanine, aspartate and glutamate metabolism | 1 | 0.5832 | 0.53922 | L-Aspartic acid cpd:C00049 |
| Phenylalanine, tyrosine and tryptophan biosynthesis | 1 | 0.60038 | 0.51019 | L-Tryptophan cpd:C00078 |
| Starch and sucrose metabolism | 1 | 0.64787 | 0.43407 | Uridine diphosphate glucose cpd:C00029 |
| Tryptophan metabolism | 1 | 0.64787 | 0.43407 | L-Tryptophan cpd:C00078 |
| Glutathione metabolism | 1 | 0.66244 | 0.41183 | Cysteinylglycine cpd:C01419 |
| Galactose metabolism | 1 | 0.66244 | 0.41183 | Uridine diphosphate glucose cpd:C00029 |
| Porphyrin and chlorophyll metabolism | 1 | 0.74913 | 0.28884 | Biliverdin cpd:C00500 |
| Aminoacyl-tRNA biosynthesis | 2 | 0.76879 | 0.26294 | L-Aspartic acid cpd:C00049;  L-Tryptophan cpd:C00078 |
| Cysteine and methionine metabolism | 1 | 0.76961 | 0.26187 | L-Aspartic acid cpd:C00049 |
| Amino sugar and nucleotide sugar metabolism | 1 | 0.78845 | 0.23769 | Uridine diphosphate glucose cpd:C00029 |
| Pyrimidine metabolism | 1 | 0.80578 | 0.21594 | Uracil cpd:C00106 |
| Purine metabolism | 1 | 0.90254 | 0.10254 | Guanine cpd:C00242 |
| **T2 vs. C2** |  |  |  |  |
| Flavonoid biosynthesis | 5 | 0.075188 | 2.5878 | Pelargonidin cpd:C05904;  Naringenin cpd:C00509;  Eriodictyol cpd:C05631;  Naringenin chalcone cpd:C06561;  Luteolin cpd:C01514 |
| Lysine biosynthesis | 2 | 0.10413 | 2.2621 | L-Aspartic acid cpd:C00049;  L-Lysine cpd:C00047 |
| Nicotinate and nicotinamide metabolism | 2 | 0.12506 | 2.0789 | L-Aspartic acid cpd:C00049;  Nicotinamide ribotide cpd:C00455 |
| Phenylalanine metabolism | 2 | 0.1469 | 1.918 | L-Phenylalanine cpd:C00079;  Phenylethylamine cpd:C05332 |
| Ascorbate and aldarate metabolism | 2 | 0.21601 | 1.5324 | Uridine diphosphate glucose cpd:C00029;  Ascorbic acid cpd:C00072 |
| Glutathione metabolism | 3 | 0.21807 | 1.523 | Cysteinylglycine cpd:C01419;  Ornithine cpd:C00077;  Ascorbic acid cpd:C00072 |
| C5-Branched dibasic acid metabolism | 1 | 0.22761 | 1.4801 | (S)-2-Acetolactate cpd:C06010 |
| Aminoacyl-tRNA biosynthesis | 6 | 0.23526 | 1.4471 | L-Histidine cpd:C00135;  L-Phenylalanine cpd:C00079;  L-Glutamine cpd:C00064;  L-Aspartic acid cpd:C00049;  L-Lysine cpd:C00047;  L-Isoleucine cpd:C00407 |
| Nitrogen metabolism | 2 | 0.2636 | 1.3333 | L-Phenylalanine cpd:C00079;  L-Glutamine cpd:C00064 |
| Alanine, aspartate and glutamate metabolism | 2 | 0.38154 | 0.96354 | L-Aspartic acid cpd:C00049;  L-Glutamine cpd:C00064 |
| Flavone and flavonol biosynthesis | 1 | 0.40398 | 0.90638 | Luteolin cpd:C01514 |
| Arginine and proline metabolism | 3 | 0.41046 | 0.89048 | Ornithine cpd:C00077;  L-Aspartic acid cpd:C00049;  L-Glutamine cpd:C00064 |
| Pyrimidine metabolism | 3 | 0.44487 | 0.80998 | L-Glutamine cpd:C00064;  Thymidine cpd:C00214;  Cytidine cpd:C00475 |
| Thiamine metabolism | 1 | 0.47662 | 0.74103 | Thiamine cpd:C00378 |
| Tropane, piperidine and pyridine alkaloid biosynthesis | 1 | 0.47662 | 0.74103 | L-Phenylalanine cpd:C00079 |
| Pentose and glucuronate interconversions | 1 | 0.47662 | 0.74103 | Uridine diphosphate glucose cpd:C00029 |
| Valine, leucine and isoleucine biosynthesis | 2 | 0.49124 | 0.71082 | (S)-2-Acetolactate cpd:C06010;  L-Isoleucine cpd:C00407 |
| Cyanoamino acid metabolism | 1 | 0.50959 | 0.67414 | L-Aspartic acid cpd:C00049 |
| Vitamin B6 metabolism | 1 | 0.50959 | 0.67414 | Pyridoxine cpd:C00314 |
| beta-Alanine metabolism | 1 | 0.54052 | 0.61523 | L-Aspartic acid cpd:C00049 |
| Phenylpropanoid biosynthesis | 2 | 0.58825 | 0.5306 | L-Phenylalanine cpd:C00079;  Coniferyl alcohol cpd:C00590 |
| Glycerolipid metabolism | 1 | 0.59671 | 0.51633 | Uridine diphosphate glucose cpd:C00029 |
| Limonene and pinene degradation | 1 | 0.59671 | 0.51633 | Perillyl aldehyde cpd:C02576 |
| Zeatin biosynthesis | 1 | 0.64611 | 0.43678 | Uridine diphosphate glucose cpd:C00029 |
| Histidine metabolism | 1 | 0.64611 | 0.43678 | L-Histidine cpd:C00135 |
| Carbon fixation in photosynthetic organisms | 1 | 0.74501 | 0.29436 | L-Aspartic acid cpd:C00049 |
| Phenylalanine, tyrosine and tryptophan biosynthesis | 1 | 0.76123 | 0.27282 | L-Phenylalanine cpd:C00079 |
| Starch and sucrose metabolism | 1 | 0.80404 | 0.21811 | Uridine diphosphate glucose cpd:C00029 |
| Galactose metabolism | 1 | 0.81655 | 0.20267 | Uridine diphosphate glucose cpd:C00029 |
| Steroid biosynthesis | 1 | 0.83926 | 0.17524 | Lathosterol cpd:C01189 |
| Glycine, serine and threonine metabolism | 1 | 0.84955 | 0.16305 | L-Aspartic acid cpd:C00049 |
| Purine metabolism | 2 | 0.87198 | 0.13698 | L-Glutamine cpd:C00064;  Guanine cpd:C00242 |
| Porphyrin and chlorophyll metabolism | 1 | 0.8846 | 0.12261 | 5-Aminolevulinic acid cpd:C00430 |
| Valine, leucine and isoleucine degradation | 1 | 0.89203 | 0.11426 | L-Isoleucine cpd:C00407 |
| Cysteine and methionine metabolism | 1 | 0.89898 | 0.1065 | L-Aspartic acid cpd:C00049 |
| Amino sugar and nucleotide sugar metabolism | 1 | 0.91158 | 0.092576 | Uridine diphosphate glucose cpd:C00029 |
| **T3 vs. C3** |  |  |  |  |
| Flavone and flavonol biosynthesis | 1 | 0.18726 | 1.6753 | Kaempferol cpd:C05903 |
| Sphingolipid metabolism | 1 | 0.28658 | 1.2497 | 3-Dehydrosphinganine cpd:C02934 |
| Glycerophospholipid metabolism | 1 | 0.47948 | 0.73506 | Phosphorylcholine cpd:C00588 |
| Galactose metabolism | 1 | 0.49305 | 0.70714 | Stachyose cpd:C01613 |
| Steroid biosynthesis | 1 | 0.51918 | 0.6555 | 24-Ethylidene lophenol cpd:C11523 |
| Amino sugar and nucleotide sugar metabolism | 1 | 0.62153 | 0.47557 | Chitobiose cpd:C01674 |
| Flavonoid biosynthesis | 1 | 0.62153 | 0.47557 | Kaempferol cpd:C05903 |
| Arginine and proline metabolism | 1 | 0.62153 | 0.47557 | Citrulline cpd:C00327 |
| Pyrimidine metabolism | 1 | 0.64123 | 0.44436 | Thymidine cpd:C00214 |

注：Pathway表示代谢通路名称；Hits表示差异代谢物命中该通路的个数；Raw p表示代谢通路富集分析的P值；-ln(p)表示P值取以e为底的负对数（负自然底对数）；Hits Cpd表示命中该通路的差异代谢物名称及KEGG ID。
