# Supplementary material for: Transcriptomics and metabolomics changes triggered by exogenous 6-benzylaminopurine in relieving epicotyl dormancy of Polygonatum cyrtonema Hua seeds
Source: Front Plant Sci. 2022 Jul 25;13:961899. doi: 10.3389/fpls.2022.961899 (PMC9358440; doi:10.3389/fpls.2022.961899)
Supplement: Supplementary Table 3 — Metabolites of P. cyrtonema seeds in different stages. [file Table_3.docx]

**Supplementary Table 3 Metabolites of *P.cyrtonema* seeds in different periods**

| Name | CAS | Class |
| --- | --- | --- |
| **T1 vs. C1** | | |
| Indole-3-carboxaldehyde | 487-89-8 | phytohormone |
| Methyl jasmonate | 39924-52-2 | phytohormone |
| Docosahexaenoic acid | 6217-54-5 | Fatty Acyls |
| Octadecanamide | 124-26-5 | Fatty Acyls |
| Pimelic acid | 111-16-0 | Fatty Acyls |
| 8,9-DiHETrE | 192461-96-4 | Fatty Acyls |
| Vitamin D2 (Ergocalciferol) | 50-14-6 | Steroids and steroid derivatives |
| Betadex | 7585-39-9 | Organooxygen compounds |
| cis-Aconitic acid | 499-12-7;585-84-2 | Organic acids and derivatives |
| Euxanthone | 529-61-3 | Xanthones |
| Vitamin A | 68-26-8;11103-57-4 | Vitamins |
| Tricholomic acid | 2644-49-7 | Carboxylic acids and derivatives |
| Biliverdin | 114-25-0 | Tetrapyrroles and derivatives |
| 11-Hydroxycanthin-6-one | 75969-83-4 | Alkaloids |
| 3-Methylindole | 83-34-1 | Alkaloids |
| Abrine | 526-31-8 | Alkaloids |
| Grossamide | 80510-06-1 | Alkaloids |
| Narciclasine | 29477-83-6 | Alkaloids |
| Protoveratrine A | 143-57-7 | Alkaloids |
| Vasicine | 6159-55-3 | Alkaloids |
| 3-O-Acetyl-11-keto-beta-boswellic acid | 67416-61-9 | Triterpenoids |
| Ganoderal A | 104700-98-3 | Triterpenoids |
| Glycyrrhetinic acid | 1449-05-4;471-53-4 | Triterpenoids |
| Homobaldrinal | 67910-07-0 | Miscellaneous |
| Butyl 4-Hydroxybenzoate | 94-26-8 | Lignans |
| Nordihydroguaiaretic acid | 500-38-9 | Lignans |
| Chalconaringenin | 73692-50-9 | flavonoids |
| Cynaroside | 5373-11-5 | flavonoids |
| Ononin | 486-62-4 | flavonoids |
| Phloretin | 60-82-2 | flavonoids |
| Rhoifolin | 17306-46-6 | flavonoids |
| sinensetin | 2306-27-6 | flavonoids |
| Isoquercitrin | 482-35-9 | flavonoids |
| 6-Hydroxyluteolin | 18003-33-3 | Flavonoids |
| Aureusidin 6-O-glucoside | 633-15-8 | Flavonoids |
| Cyanidin | 528-58-5 | Flavonoids |
| Cyanin | 2611-67-8;20905-74-2 | Flavonoids |
| Dalbergioidin | 30368-42-4 | Flavonoids |
| Formononetin 7-O-glucoside-6''-O-malonate | 34232-16-1 | Flavonoids |
| Kaempferol 3-O-beta-sophoroside | 19895-95-5 | Flavonoids |
| Linarin | 480-36-4 | Flavonoids |
| Maltol | 118-71-8 | Flavonoids |
| Puerarin;Puararin | 3681-99-0 | Flavonoids |
| 7,3'-Dihydroxy-4'-methoxy-8-methylflavan | 87733-81-1 | Flavonoids |
| Cyanidin 3-O-3'',6''-O-dimalonylglucoside | 171828-60-7 | Flavonoids |
| Geniposide | 24512-63-8 | Iridoids |
| Uracil | 66-22-8 | Nucleotide and its derivates |
| Guanosine 3',5'-cyclic monophosphate | 7665-99-8 | Nucleotide and its derivates |
| 7-(4-Hydroxyphenyl)-1-phenyl-4-hepten-3-one | 100667-52-5 | Phenols |
| Cannabinol | 521-35-7 | Phenols |
| DL-alpha-Tocopherylacetate | 7695-91-2 | Phenols |
| Kakuol | 18607-90-4 | Phenols |
| Methyl benzoate | 93-58-3 | Phenols |
| Myricanone | 32492-74-3 | Phenols |
| Crocin | 42553-65-1 | Diterpenoids |
| Cinnamyl cinnamate | 122-69-0 | Phenylpropanoids |
| Xanthotoxin | 298-81-7 | Phenylpropanoids |
| Cinnamic acid | 140-10-3 | Phenylpropanoids |
| Allysine(6-Oxo DL-Norleucine) | 6665-12-9 | Amino acid and derivatives |
| 4-Hydroxyproline | 51-35-4 | Amino acid and derivatives |
| L-Tryptophan | 73-22-3 | Amino acid and derivatives |
| **T2 vs. C2** |  |  |
| Methylimidazole acetaldehyde | 19639-03-3 | Azoles |
| Methyl jasmonate | 39924-52-2 | phytohormone |
| 9-OxoODE | 54232-59-6 | Fatty Acyls |
| Lathosterol | 80-99-9 | Steroids and steroid derivatives |
| Stigmasterol | 83-48-7 | Steroids and steroid derivatives |
| Progesterone | 57-83-0 | Steroids |
| Perillyl aldehyde | 2111-75-3 | Prenol lipids |
| Pseudouridine 5'-phosphate | 1157-60-4 | Organooxygen compounds |
| 5-Aminolevulinate | 106-60-5 | Organic acids |
| Leukoaminochrome | 29539-03-5 | Indoles and derivatives |
| (+)-Pteryxin | 17944-23-9;13161-75-6 | Coumarins |
| Bergaptol | 486-60-2 | Coumarins |
| Ascorbic acid | 50-81-7 | Vitamins |
| Pyridoxine | 65-23-6 | Vitamins |
| Thiamine | 59-43-8 | Vitamins |
| D-Xylulose | 551-84-8 | Carbohydrates |
| (2E)-Decenoyl-ACP | 52-52-8 | Carboxylic acids and derivatives |
| Asp-Phe methyl ester;Aspartame | 22839-47-0 | Carboxylic acids and derivatives |
| D-Glutamine | 5959-95-5 | Carboxylic acids and derivatives |
| N-Acetyl-L-histidine | 2497-02-1 | Carboxylic acids and derivatives |
| Chimonanthine | 5545-89-1 | Alkaloids |
| Vincamine | 1617-90-9 | Alkaloids |
| Azadirachtin | 11141-17-6 | Triterpenoids |
| Cyclomusalenone | 30452-60-9 | Triterpenoids |
| Glycyrrhetinic acid | 1449-05-4;471-53-4 | Triterpenoids |
| Baldrinal | 18234-46-3 | Miscellaneous |
| Ethyl isovalerate | 108-64-5 | Miscellaneous |
| Pregabalin | 148553-50-8 | Miscellaneous |
| 2'-O-Methyladenosine | 2140-79-6 | Purine nucleosides |
| Daidzin | 552-66-9 | flavonoids |
| Luteolin | 491-70-3 | flavonoids |
| Isoquercitrin | 482-35-9 | flavonoids |
| (-)-Glyceollin II | 67314-98-1 | Flavonoids |
| 2'-Hydroxybiochanin A | 32884-35-8 | Flavonoids |
| 2'-Hydroxydaidzein | 7678-85-5 | Flavonoids |
| 2'-O-Methylisoliquiritigenin | 112408-67-0 | Flavonoids |
| Cyanidin-3-O-rutinoside chloride | 18719-76-1 | Flavonoids |
| Delphinidin 3-O-(6''-O-malonyl-beta-D-glucoside) |  | Flavonoids |
| Formononetin | 485-72-3 | Flavonoids |
| Glyceollin III | 61080-23-7 | Flavonoids |
| Isoetin | 1621-84-7 | Flavonoids |
| Kievitone | 40105-60-0 | Flavonoids |
| Kievitone hydrate | 62682-11-5 | Flavonoids |
| Luteone | 41743-56-0 | Flavonoids |
| Malonylglycitin | 137705-39-6 | Flavonoids |
| Naringenin chalcone | 25515-46-2 | Flavonoids |
| Pectolinarin | 28978-02-1 | Flavonoids |
| Pelargonidin | 7690-51-9;134-04-3 | Flavonoids |
| Peonidin-3-glucoside | 68795-37-9 | Flavonoids |
| Quercetin 3-O-malonylglucoside;Quercetin 3-(6''-malonyl-glucoside) | 96862-01-0 | Flavonoids |
| Sulfuretin | 120-05-8 | Flavonoids |
| 2,3-Dihydro-2-phenyl-4H-benzopyran-4-one | 487-26-3 | Flavonoids |
| Cyanidin 3,5,3'-tri-O-glucoside | 88110-66-1 | Flavonoids |
| 3-Methylxanthine | 1076-22-8 | Nucleotide and its derivates |
| Cytidine | 65-46-3 | Nucleotide and its derivates |
| Benzaldehyde | 100-52-7 | Phenols |
| Hamamelitannin | 469-32-9 | Phenols |
| Benzocaine | 94-09-7 | Benzene and substituted derivatives |
| Phenethylamine | 64-04-0 | Benzene and substituted derivatives |
| Dulcoside A | 64432-06-0 | Diterpenoids |
| (1R)-(-)-Menthyl acetate | 2623-23-6 | Monoterpenoids |
| Queuine | 72496-59-4 | Pyrrolopyrimidines |
| Coniferyl alcohol | 458-35-5;32811-40-8 | Phenylpropanoids |
| Cinnamic acid | 140-10-3 | Phenylpropanoids |
| alpha-Cyperone | 473-08-5 | Sesquiterpenoids |
| Lactupicrin | 65725-11-3 | Sesquiterpenoids |
| Linderalactone | 728-61-0 | Sesquiterpenoids |
| 5-Aminovaleric acid | 660-88-8 | Amino acid and derivatives |
| Isoleucine | 443-79-8 | Amino acid and derivatives |
| L-Isoleucine | 73-32-5 | Amino acid and derivatives |
| L-Kynurenine | 343-65-7;2922-83-0 | Amino acid and derivatives |
| L-Lysine | 56-87-1 | Amino acid and derivatives |
| L-Norleucine | 327-57-1 | Amino acid and derivatives |
| N6-Acetyl-L-lysine | 692-04-6 | Amino acid and derivatives |
| 4-Aminobutyric acid | 56-12-2 | Amino acid and derivatives |
| 5-Hydroxylysine | 1190-94-9 | Amino acid and derivatives |
| L-Glutamine | 56-85-9 | Amino acid and derivatives |
| L-Histidine | 71-00-1 | Amino acid and derivatives |
| L-Ornithine | 70-26-8 | Amino acid and derivatives |
| L-Phenylalanine | 63-91-2 | Amino acid and derivatives |
| **T3 vs. C3** |  |  |
| N6-isopentenyladenosine | 7724-76-7 | phytohormone |
| Methyl jasmonate | 39924-52-2 | phytohormone |
| Pentadecanoic acid | 1002-84-2 | Fatty Acyls |
| Citrostadienol | 474-40-8 | Steroids |
| Guggulsterone E&Z | 95975-55-6 | Steroids |
| Resibufogenin | 465-39-4 | Steroids |
| 3-Dehydrosphinganine | 16105-69-4 | Organooxygen compounds |
| Stachyose | 10094-58-3;470-55-3 | Organooxygen compounds |
| N,N'-diacetylchitobiose | 35061-50-8 | Organooxygen compounds |
| 1H-Indole-2,3-dione | 91-56-5 | Indoles and derivatives |
| Norathyriol | 3542-72-1 | Xanthones |
| Bergamotine | 7380-40-7 | Coumarins |
| Decursin | 5928-25-6 | Coumarins |
| Maltotetraose | 34612-38-9 | Carbohydrates |
| Hydroxypropanedioic acid | 80-69-3 | Carboxylic acids and derivatives |
| Baptifoline | 732-50-3 | Alkaloids |
| Cinchonine | 118-10-5 | Alkaloids |
| Nicotinamide | 98-92-0 | Alkaloids |
| Riddelline | 23246-96-0 | Alkaloids |
| Pristimerin | 1258-84-0 | Triterpenoids |
| Alliin | 556-27-4 | Miscellaneous |
| Panaxynol | 81203-57-8 | Miscellaneous |
| Torachrysone 8-O-glucoside | 64032-49-1 | Miscellaneous |
| Shikonin | 517-89-5 | Quinones |
| 9-Riburonosyladenine | 553592 | Purine Nucleosides |
| 1,2,5,6-Tetrahydro-4H-pyrrolo[3,2,1-ij]quinolin-4-one | 57369-32-1 | Quinolines and derivatives |
| Apiin | 26544-34-3 | flavonoids |
| Narcissoside | 604-80-8 | flavonoids |
| Vitexin | 3681-93-4 | flavonoids |
| Isoquercitrin | 482-35-9 | flavonoids |
| Afzelechin-(4alpha->8)-afzelechin | 101339-37-1 | Flavonoids |
| Bracteatin | 3260-50-2 | Flavonoids |
| Chrysin dimethylether | 21392-57-4 | Flavonoids |
| Chrysoeriol 7-apiosylglucoside | 33579-63-4 | Flavonoids |
| Epicatechin-(4beta->8)-ent-epicatechin | 82262-99-5 | Flavonoids |
| Ficine | 2520-36-7 | Flavonoids |
| Genistein 8-C-glucoside | 66026-80-0 | Flavonoids |
| Hydrangenol | 480-47-7 | Flavonoids |
| Hypolaetin | 27696-41-9 | Flavonoids |
| Isoficine | 2255-62-1 | Flavonoids |
| Kaempferol | 520-18-3 | Flavonoids |
| Karanjin | 521-88-0 | Flavonoids |
| Myricitrin;Myricetin 3-O-rhamnoside (Myricitrin) | 17912-87-7 | Flavonoids |
| Saponarin | 20310-89-8 | Flavonoids |
| Trifolirhizin | 6807-83-6 | Flavonoids |
| Vicenin 2 | 23666-13-9 | Flavonoids |
| 2',3,5,7-Tetrahydroxyflavone | 480-15-9 | Flavonoids |
| Beta-Tocopherol | 148-03-8 | Phenols |
| Miltirone | 27210-57-7 | Diterpenoids |
| O-Phosphocholine | 3616-04-4;  107-73-3 | Cholines |
| Zeranol | 26538-44-3 | Macrolides and analogues |
| Carbendazim | 10605-21-7 | Benzimidazoles |
| Imperatorin | 482-44-0 | Phenylpropanoids |
| Cinnamic acid | 140-10-3 | Phenylpropanoids |
